# Editorial for Special Issue: “Thin Films Based on Nanocomposites”

**DOI:** 10.3390/nano12193301

**Published:** 2022-09-22

**Authors:** Marcela Socol, Nicoleta Preda

**Affiliations:** National Institute of Materials Physics, 405A Atomistilor Street, P.O. Box MG-7, 077125 Magurele, Romania

Nanocomposites gained great attention from both fundamental scientific research and technological application perspectives emerging as a fascinating class of advanced functional materials, that can find applications in various areas such as electronics, energy, environmental protection, healthcare, etc. In this framework, this special issue includes sixteen research papers (fifteen articles and one review) covering the preparation and characterisation of nanocomposites for a wide range of applications, which extends from optoelectronic devices to the biomedical field.

Metal oxides and their composites turned out to be widely explored due to their structural, morphological, physicochemical, optical and electrical features that can be relatively easily tuned during the preparation process, the final compounds having properties in line with the targeted applications ranging from (opto-) electronic devices [1,2,3,4] to antibacterial materials [5], photocatalysts [6] or wearable sensors [7].

Molkenova et al. developed an easy and scalable solution-based process for fabricating transparent TiO_x_ and Eu-doped TiO_x_ thin films on glass substrates, these metal oxide layers lowering significantly the transmittance of destructive UV radiation, a very useful feature for the protection of photovoltaic devices [1]. Hu et al. analysed the effects of the experimental parameters involved in the deposition of amorphous Indium–Gallium–Zinc Oxide (a-IGZO) films by radio frequency (RF) magnetron sputtering on their properties and further, on the performance of thin-film transistors developed with such a-IGZO films [2]. Min et al. compared the performances of memristor devices using Indium–Gallium–Zinc Oxide (IGZO) or nitride IGZO (IGZO:N) nanocomposite films as resistive switching (RS) layers and revealed that an improvement of the memristive switching properties for potential synaptic electronics is achieved when microwave-assisted nitridation technology on the solution-derived metal oxide-based RS layer is involved [3]. Boukhoubza et al. synthesized ZnO nanorods/graphene oxide (GO) nanocomposites by a hydrothermal process evaluating the effect of the graphene oxide amount on the morphological, structural and optical properties of the nanocomposites, the results being promising for the development of optoelectronic devices based on graphene derivates [4]. Zgura et al. used a “green” approach for synthesizing Ag nanoparticles, ZnO nanoparticles and AgZnO nanocomposites in the presence of aqueous vegetal extracts from *Caryophyllus aromaticus* L. (cloves) and *Citrus reticulata* L. (mandarin) peels, the phyto-based composites proving biocidal activity against *Staphylococcus aureus*, biocompatibility on human fibroblast BJ cells and no damage on the human red blood cells [5]. Enculescu et al. prepared ZnO and TiO_2_ nanotubes by a three-step fabrication process (electrospinning for poly(methyl methacrylate) (PMMA) nanofibers template, RF magnetron sputtering for ZnO or TiO_2_ layers deposition and calcination for removing the organic template), the nanostructures displayed high photocatalytic activity against Rhodamine B under white light irradiation, a useful characteristic for water splitting and water purification [6]. Beregoi et al. fabricated core–double-shell nylon-ZnO/polypyrrole electrospun nanofibers by a three-step fabrication process (electrospinning for nylon fibre core, sol-gel for ZnO shell layer and electrodeposition for polypyrrole (PPy) film), the nanomaterials being highly transparent and flexible, embedding a semiconducting heterojunction are relatively easily integrated into functional platforms for wearable sensors, intelligent clothes, etc. [7].

Some studies are focused on nanocomposites based on polymer and metal oxides that are promising for applications, which require materials with high elastic strength [8], high modulus of elasticity and yield strength and resistance to thermal shrinkage [9] or high antibacterial activity [10].

Kumari et al. investigated nanocomposites films based on glycolic acid-grafted chitosan (GA-g–CS) and Pt–Fe_3_O_4_ nanoparticles with enhanced elastic properties, the incorporation of Pt–Fe_3_O_4_ nanoparticles in the chitosan favouring the formation of intermolecular bonds, which allows the rotation of the polymer chains [8]. Lee et al. prepared nanocomposites films based on high-density polyethylene (HDPE) and boehmite (BA) nanoparticles, unmodified or modified with vinyltrimethoxysilane (vBA), by a melt-blending technique; further, the films were irradiated with electrons for improving their mechanical and thermal properties through the increase of the interfacial adhesion between the modified-nanofiller and HDPE matrix, which in its turn is related to the formation of strong covalent bonds between HDPE and hydroxyl groups-BA [9]. Zhou et al. obtained nanocomposites films based on starch and poly(butylene adipate-co-terephthalate) (PBAT) and AgNPs@SiO_2_ particles by the melt-blending and blowing technique, these films have high inhibition efficiency against both Staphylococcus aureus and Escherichia coli, preliminary packaging studies carried on fresh fruits revealing that these materials inhibit their spoilage; thus, having great potential for commercial food packaging [10]. 

Several studies are dedicated to thin film composites with components such as carbon nanostructures, polymers or semiconductors for potential applications in multi-functional temperature resistance aerospace structures [11], (opto-) electronic devices [12], scintillation detectors [13] or photovoltaic cells [14,15].

Che et al. fabricated carbon nanotube film-reinforced mesophase pitch-based carbon (CNTF/MPC) nanocomposites by hot-pressing carbonization, the mechanical properties, heat resistance and electrical and thermal conductivities of CNTF/MPC being enhanced in comparison to the pristine carbon nanotube film [11]. Shan et al. prepared boron-doped Si nanocrystals embedded in the amorphous SiC films by plasma-enhanced chemical vapour deposition, the electrical properties of these nanomaterials being strongly related to the Si/C ratio [12]. Děcká et al. deposited CsPbBr_3_ thin films on scintillating GGAG:Ce (Gd_2.985_Ce_0.015_Ga_2.7_Al_2.3_O_12_) wafer by spin-coating and studied their radioluminescence (RL) response, a synergic effect consisting in the enhancement of RL intensity and the preservation of the ultrafast CsPbBr_3_ decay being emphasized and proving the potential of these composites in time-of-flight positron emission tomography (TOF-PET) field [13]. Lu et al. obtained FeS_2_ thin films by electrochemical deposition and revealed that the amorphous precursor films become crystalline after sulfurization sintering, further to a heterojunction based on FeS_2_ and poly(3-hexylthiophene-2,5-diyl) (P3HT) being developed [14]. Socol et al. deposited thin films from blends based on a conjugated polymer, poly[2,5-(2-octyldodecyl)-3,6-diketopyrrolopyrrole-alt-5,5-(2,5-di(thien-2-yl)thieno [3,2-b]thiophene)] (DPP-DTT) and fullerene C_60_ by Matrix Assisted Pulsed Laser Evaporation (MAPLE) and evaluated the influence of some experimental parameters on their properties, further to the structures developed on these films evidencing a photovoltaic cell behaviour [15].

Socol and Preda reviewed the progress achieved in the last ten years in the field of photovoltaic cells based on hybrid nanocomposite thin films deposited by spin-coating and MAPLE [16]. The overview summarises the organic compounds (conducting polymers and metal phthalocyanines as *p*-type materials and fullerene derivatives and non-fullerene compounds as *n*-type materials) and inorganic semiconductor nanostructures (metal oxide, chalcogenides and silicon) frequently used in the preparation of the organic:inorganic mixtures for fabricating hybrid layers for the photovoltaic devices. In addition, the influence of various experimental parameters on the hybrid solar cell efficiency is throughout analysed for designing hybrid composite films with adequate properties in order to enhance the device performance.

As demonstrated in this Special Issue, it is essential to design and develop nanocomposites with tailored properties in order to expand the range of their potential applications in the years to come.
